# Soledad Sepúlveda or the importance of being an REDLARA embryologist

**DOI:** 10.5935/1518-0557.20160048

**Published:** 2016

**Authors:** Maria Teresa Urbina

**Affiliations:** 1 President of Latin American Network of Assisted Reproduction (REDLARA), Uruguay; 2 Clinica El Avila, Caracas, Venezuela; 3 Unifertes Fertility Center, Caracas, Venezuela

It is with deep sadness that we write that Dr. Soledad Sepúlveda passed away earlier this summer (or give date). Sole was among the earliest pioneers in Assisted Reproductive Technologies in Latin America and her premature departure has caused great sadness for all of us who loved and admired her.

After Louise Brown's birth in 1978, the first IVF birth in Latin America succeeded in 1985. Ten years later REDLARA was founded, and a remarkable founder member was Soledad Sepúlveda (Sole).

The field of human IVF has matured progressively in its development. It is well recognized that the basis for human IVF success at its beginning can be attributed to the first reports of hamster sperm capacitation from Yanagimachi and Chang (Yanagimachi & Chang 1963; 1964; Bavister 2002). Hamster IVF was done in microdroplets of media, under oil, in low oxygen pressure. But, the human embryo exhibits a considerable degree of plasticity, which enables it to develop under sub-optimal conditions (without oil, low oxygen pressure, adequate temperature and pH). It is the most resilient embryo of all mammalian species studied to date (Gardner & Lane, 2012). Having to adapt to sub-optimal conditions came at the cost of impaired viability and compromised potential outcomes (Gardner & Lane, 2012). Nowadays, IVF success has significantly improved as shown by the live birth rates from initiated cycle from less than 1% in the beginning to over 41% in women under 35 years (exceeding 60% in some programs) (McCulloh 2012, Zegers-Hochschild *et al*., 2016).

What can success be attributed to? New media, new recombinant gonadotropins, new equipment, new procedures, quality control, regulations, optimal culture conditions and Soledad Sepúlveda!

Sole belonged to that generation of embryologists who learned in universities how to do hamster IVF and knew who Yanagimachi is. Very particular characteristics, because there are only a few formal teaching and skill examination programs in place for a specialty in ART (Cohen *et al.,* 2012). Sole studied Biology at Universidad de Chile under the mentorship of renowned Professor Luis Izquierdo, where she earned her Master and Doctoral Degrees, she also held post-doctoral studies on reproductive biology and reproductive immunology. She used Evidence Based Medicine to help laboratories have optimal culture conditions and have very specialized embryologists. Sole showed us that good clinical outcomes depend on well-trained embryologists, who perform gamete and embryo handling; culture procedures; set and maintain quality standards; register changes, incidents and unexpected events, and corrective measures. Sole made us do IVF in microdroplets media under oil, with optimal culture conditions, low oxygen pressure, adequate temperature and pH... as she learned in the university and from her continuous education. Sole made embryologists think like they were embryos! Not only that, she tracked the individual clinician performances to demonstrate that IVF success was also dependent on the skills of fertility physicians.

As a product of her work on IVF, Sole published more than 30 scientific papers and book chapters (Sepúlveda, 2011) and contributed to writing and editing the REDLARA guidelines (RED Latinoamericana de Reproducción Asistida 2015; 2016).

Solidarity and generosity were other key factors to Sole's success. She shared her knowledge with all the fertility centers where she was required. Dozens of REDLARA embryologists were Hands-on trained by her. She spent a huge portion of her life promoting excellence in reproductive medicine through education. As a consultant, she set many IVF centers on track to having high standards. Sole reviewed all the international quality standards for IVF and helped develop REDLARA Accreditation, Certification, and Continuous Education Programs. If that was not enough, Sole was the first embryologist to be a Regional Director of REDLARA and organized several workshops inviting relevant international IVF scientists and embryologists as speakers. Our friend Sole participated with great enthusiasm in the developing of every REDLARA program, step by step, assuring quality and increasing IVF success to benefit patient outcomes. She attended each REDLARA workshop, surrounding us with her joy, laughter, and happiness, and united us with her great love. Sole worked in her laboratory until her last days and is resting now, but her extraordinary legacy in REDLARA will last forever!
